# RNA-seq analysis reveals the role of red light in resistance against *Pseudomonas syringae* pv. *tomato* DC3000 in tomato plants

**DOI:** 10.1186/s12864-015-1228-7

**Published:** 2015-02-25

**Authors:** You-Xin Yang, Meng-Meng Wang, Yan-Ling Yin, Eugen Onac, Guo-Fu Zhou, Sheng Peng, Xiao-Jian Xia, Kai Shi, Jing-Quan Yu, Yan-Hong Zhou

**Affiliations:** Department of Horticulture, Zijingang Campus, Zhejiang University, Yuhangtang Road 866, Hangzhou, 310058 P. R. China; Key Laboratory of Horticultural Plants Growth, Development and Quality Improvement, Agricultural Ministry of China, Zijingang Road 866, Hangzhou, 310058 P. R. China; Philips Research China, No. 9 Lane 888 Tian Lin Road, Shanghai, 200233 P. R. China; Philips Research Europe, High Tech Campus 34, 5656 AE Eindhoven, Netherlands

## Abstract

**Background:**

Plants attenuate their responses to a variety of bacterial and fungal pathogens, leading to higher incidences of pathogen infection at night. However, little is known about the molecular mechanism responsible for the light-induced defence response; transcriptome data would likely facilitate the elucidation of this mechanism.

**Results:**

In this study, we observed diurnal changes in tomato resistance to *Pseudomonas syringae* pv. *tomato* DC3000 (*Pto* DC3000), with the greatest susceptibility before midnight. Nightly light treatment, particularly red light treatment, significantly enhanced the resistance; this effect was correlated with increased salicylic acid (SA) accumulation and defence-related gene transcription. RNA-seq analysis revealed that red light induced a set of circadian rhythm-related genes involved in the phytochrome and SA-regulated resistance response. The biosynthesis and signalling pathways of multiple plant hormones (auxin, SA, jasmonate, and ethylene) were co-ordinately regulated following *Pto* DC3000 infection and red light, and the SA pathway was most significantly affected by red light and *Pto* DC3000 infection. This result indicates that SA-mediated signalling pathways are involved in red light-induced resistance to pathogens. Importantly, silencing of *nonexpressor of pathogensis-related genes 1* (*NPR1*) partially compromised red light-induced resistance against *Pto* DC3000. Furthermore, sets of genes involved in redox homeostasis (*respiratory burst oxidase homologue*, *RBOH*; *glutathione S-transferases*, *GSTs*; *glycosyltransferase*, *GTs*), calcium (*calmodulin*, *CAM*; *calmodulin-binding protein*, *CBP*), and defence (*polyphenol oxidase*, *PPO*; *nudix hydrolase1*, *NUDX1*) as well as transcription factors (*WRKY18*, *WRKY53*, *WRKY60*, *WRKY70*) and cellulose synthase were differentially induced at the transcriptional level by red light in response to pathogen challenge.

**Conclusions:**

Taken together, our results suggest that there is a diurnal change in susceptibility to *Pto* DC3000 with greatest susceptibility in the evening. The red light induced-resistance to *Pto* DC3000 at night is associated with enhancement of the SA pathway, cellulose synthase, and reduced redox homeostasis.

**Electronic supplementary material:**

The online version of this article (doi:10.1186/s12864-015-1228-7) contains supplementary material, which is available to authorized users.

## Backgrounds

Plants have evolved intricate mechanisms for perceiving external signals, thereby enabling an optimal response to biotic and abiotic stimuli. A number of signalling pathways with roles in regulating the response to pathogens have been defined. Upon infection by microbial pathogens, plants activate a multitude of defence responses to combat the attacking intruders [1]. Salicylic acid (SA), jasmonate (JA), and ethylene (ET) contribute to responses to biotic stresses by influencing various signalling pathways with complex networks of synergistic and antagonistic interactions [2]. The plant-pathogen interaction results in a burst of reactive oxygen species (ROS), the rapid induction of a hypersensitive response (HR), and the expression of pathogenesis-related (*PR*) genes at the infection site [3]. The plants also develop systemic acquired resistance (SAR) in uninfected tissues of plants following an HR [4,5]. SA is a signalling molecule that acts during SAR development. Meanwhile, the responses of plants to pathogens are largely dependent on environmental factors such as light, temperature, and water. For example, low temperature suppresses the resistance of rice (*Oryza sativa*) plants to infection by *Magnaporthe grisea* [6]. A recent study revealed an antagonistic interaction between SAR and ABA induced by abiotic stress factors [7].

Light is one of the major external factors that influence plant growth and development. Recent studies have revealed that light is also required for establishing an efficient response in several plant-pathogen interactions [8-10]. Plants have evolved several types of photoreceptors to perceive and respond to the quantity and quality of light. These photoreceptors comprise the red/far-red light-absorbing phytochromes, the UV-A and blue light-absorbing cryptochromes, and phototropins [11]. Plants have increased defence capability against pathogen attack during the daytime, which is attributable to the availability of a prolonged light period during the early stages of the plant-pathogen interaction [9]. Both SAR and HR are light dependent and require the PHYA and PHYB light receptors [9,12]. In the dark, plants attenuate their responses to a variety of bacterial and fungal pathogens, leading to a higher incidence of infection at night [13,14]. Moreover, the induced emissions of volatile compounds in the herbivore resistance responses are light dependent [15].

Several studies have contributed to our understanding of induced defence responses to light stimuli at the molecular level. The accumulation of SA, the induction of phenylalanine ammonia-lyase (PAL), and the expression of the pathogenesis-related protein (*PR1*) were light dependent in *Arabidopsis* plants inoculated with an avirulent strain of *Pseudomonas syringae* [16]. Microarrays have recently been used to identify differentially expressed genes in *Arabidopsis* in response to light at low temperature or under wound stress [17,18]. A specific role of light has been implicated in the responses of plants to pathogen attack [19], but the specific role of light in global gene expression in the defence response has remained elusive.

We previously demonstrated that red light induced resistance to powdery mildew more effectively than other types of light, a process with enhancement of the SA-mediated defence pathway [20]. In this study, we examined the diurnal changes that affect resistance and determined whether nightly light exposure could trigger the defence against *Pseudomonas syringae* pv. *tomato* strain DC3000 (*Pto* DC3000) in tomato plants. Subsequently, RNA-seq was performed to detect whole genome expression changes. The results demonstrated that supplementary red light at night can enhance the plant defence against pathogen infection; the effect of red light was associated with a set of differentially expressed genes, particularly those involved in the circadian rhythm, photosynthesis, ROS, calcium signalling, and hormone regulation. The possible roles of these genes in the induced resistance to *Pseudomonas syringae* pv. *tomato* DC3000 (*Pto* DC3000) mediated by red light are discussed.

## Methods

### Plant materials, virus-induced gene silencing (VIGS) constructs and Agrobacterium-mediated virus infection

Tomato (*Solanum lycopersicum* L, cv. Ailsa Craig) seeds were sown in trays filled with a mixture of peat and vermiculite (2:1, v/v) and placed in growth chambers at a temperature of 25/19°C (day/night) with a photoperiod of 12 h of light (8:00 AM to 8:00 PM), a photosynthetic photon flux density (PPFD) of 200 μmol m^−2^ s^−1^ from fluorescent tubes, and a relative humidity (RH) of 70%. The seedlings were watered daily and fertilised with Hoagland nutrition solution once per week.

VIGS was performed using the bipartite tobacco rattle virus (TRV) vectors, pTRV1 and pTRV2, as previously described [21]. Fragments from tomato nonexpressor of PR1 *(NPR1)*, protein inhibitor I/II (*PI I and II*) cDNAs were PCR-amplified using the primers shown in Additional file 1: Table S1. Restriction sites were added to the 5′-ends of the forward and reverse primers for cloning into the pTRV2 vector. The amplified fragment was digested with SacI and XhoI and ligated into the same sites of pTRV2. The resulting plasmid was transformed into *Agrobacterium tumefaciens* GV3101. *Agrobacterium*-mediated virus infection was performed as previously described [21]. pTRV*-PI I/II* was an equal mix of pTRV-*PI I* and pTRV-*PI II*. Plants were then kept at 23/21 ^o^C under 120 μmol m^−2^ s^−1^ PPFD for 30 d before they were used for the experiments.

### Nighttime light treatment

To determine the diurnal changes in disease resistance, tomato plants were inoculated with *Pseudomonas syringae* pv. *tomato* strain DC3000 (DC3000) at the four-leaf stage every 4 h on one day, at 0:00, 4:00, 8:00, 12:00, 16:00, and 20:00. To investigate the effects of different types of light on defence, tomato seedlings at the four-leaf stage were exposed to nightly light treatments from 8:00 PM to 8:00 AM using purple light (P) with a maximum intensity at 394 nm, blue light (B) with a maximum intensity at 452 nm, green light (G) with a maximum intensity at 522 nm, yellow light (Y) with a maximum intensity at 594 nm, or red light (R) with a maximum intensity at 660 nm (Additional file 2: Figure S1). The P, B, G, Y, and R lights were provided by light-emitting photodiodes (LEDs, T8-1200 mm-15 W, Qiushi Co., China). Plants placed under a dark environment at night (8:00 PM to 8:00 AM) were used as controls. The intensity of the supplemental light was set at 20 μmol m^−2^ s^−1^ PPFD at the level of the canopy. Three days after light treatment, half of the plants were inoculated with DC3000, and the other plants were treated with MgCl_2_ as a mock treatment on the fourth day at 10:00 AM. Leaf samples for gene transcript and biochemical analysis were collected at 0, 1, 2, and 3 days post-inoculation (dpi).

### Growth of the pathogen DC3000 and inoculation of tomato plants

DC3000 was grown in King’s B medium containing rifampicin (50 mg ml^−1^) at 28°C as previously described [22]. One day prior to inoculation, a single bacterial colony was cultured at 28°C with shaking until the log phase. Then, the cells were collected by centrifugation at 4,000 *g* for 10 min and resuspended in 10 ml of 10 mM MgCl_2_. The inoculation was performed by dipping the entire leaf into the bacterial suspension (OD600 = 0.1 in 10 mM MgCl_2_) for 2 to 3 seconds to ensure that the leaf surfaces were coated with the bacterial suspension. Control mock inoculations were performed using MgCl_2_ buffer only. The plants were then placed at 90 to 100% RH for the first 24 h and then at approximately 70% RH for the rest of the experimental period. The leaves were photographed at 6 d after DC3000 infection. The determination of bacterial growth colony-forming units (CFU) in plant was performed as described [23]. Three 0.79 cm^2^ leaf discs from each leaf were pooled as one replicate and the leaf discs were homogenised in 1 ml of 10 mM MgCl_2_. Bacterial growth numbers were determined by plating appropriate dilutions in King’s B medium with rifampicin (50 μg ml^−1^). CFU were counted after incubation for 48 h at 28°C. All pathogen experiments were repeated twice, and similar results were obtained.

### Characterisation of microscopic disease lesions

Trypan blue staining was performed at 3 days after DC3000 inoculation as described by [24]. Lactophenol-trypan blue infection (10 ml of lactic acid, 10 ml of glycerol, 9.3 g of phenol, and 10 mg of trypan blue dissolved in 10 ml of distilled water) was used to study the development of the pathogen in stained whole leaves. Whole leaves were boiled for approximately 2–4 min in the stain solution and then decolorised in chloral hydrate (25 g of chloral hydrate dissolved in 10 ml of distilled water) for 2–3 h. The leaves were stored in chloral hydrate and photographed under a compound microscope.

### Measurement of gas exchange, chlorophyll fluorescence parameters, chlorophyll content and electrolyte leakage

The gas exchange parameter as the light-saturated rate of CO_2_ assimilation (*A*_sat_) was measured on the third fully expanded leaves using an infrared gas analyser (Li-COR 6400; Li-COR, Lincoln, NE, USA) at 3 days after DC3000 inoculation. The measurements were performed from 9:00 AM to 11:00 AM under the following conditions: 25°C, 380 μmol mol^−1^ CO_2_, 70% RH, and 1000 μmol m^−2^ s^−1^ PPFD. Chlorophyll (Chl) fluorescence was measured on the third fully expanded leaves after 30 min of dark adaptation using an imaging pulse amplitude-modulated (PAM) fluorometer (IMAG-MAXI; Heinz Walz, Effeltrich, Germany). The intensities of the actinic light and saturating light settings were 280 μmol m^−2^ s^−1^ and 2500 μmol m^−2^ s^−1^ PPFD, respectively. The maximum quantum yield of PSII (*Fv/Fm*) was measured and calculated as described by Huang et al. [25]. Leaf chlorophyll (Chl *a* and Chl *b*) was extracted in 80% acetone, and the contents (μg g^−1^ FW) were determined spectrophotometrically according to Lichtentaler and Wellburn [26]. Electrolyte leakage was measured in leaves at 3 days after DC3000 inoculation as previously described [27]. The conductivity was measured using an electroconductivity meter (DDS-11A, Beijing, China).

### Determination of glutathione contents in the leaves

The glutathione content was determined according to [28] using an enzymatic recycling method. Leaf tissue (0.3 g) was homogenised in 2 ml of 6% metaphosphoric acid containing 2 mM EDTA. The homogenates were then centrifuged at 4°C for 10 min at 14,000 *g*. Total glutathione was sequentially oxidised by 5,5′-dithiobis-2-nitrobenzoic acid (DTNB) and reduced by NADPH in the presence of GR. Oxidised glutathione (GSSG) was assayed by derivatising reduced glutathione (GSH) with 2-vinylpyridine. The GSH content was then calculated by deducting GSSG from the total glutathione.

### Determination of SA

The extraction and quantification of free and conjugated SA were measured as described previously [20] with some modification. Briefly, leaf samples (0.3 g) were ground in 3 ml of 90% methanol and centrifuged. The combined supernatants were dried under vacuum at 40°C, and the obtained residue was dissolved in 3 ml of distilled water at 80°C for 10 min. For the assay of free SA, 1 ml of supernatant was extracted with 2.5 ml of ethylacetate-cyclopentane (1:1, v/v) and 50 μl of 10 N HCl and subsequently dried under nitrogen. The residues were dissolved in 1 ml of 20% (v/v) methanol in 20 mM sodium acetate buffer (pH 5.0) and subjected to HPLC (LC-10AS; Shimadzu, Kyoto, Japan). The amount of SA in a 20 μl sample was determined with a HPLC spectrofluorescence detector (RF-10AXL; Shimazu, Tokyo, Japan) at an excitation wavelength of 295 nm and an emission wavelength of 370 nm. The flow rate was 1.0 ml min^−1^, the solvent was 20% (v/v) methanol/20 mM sodium acetate buffer (pH 5.0), and the ODS column (C18, 4.6 × 250 mm) was maintained at 35°C. For the determination of SA glucosides (SAG), 1 ml of supernatant was incubated with 1 ml of β-glucosidase (3 U ml^−1^) at 37°C for 6 h, and the SAG level was then determined as in the assay of free SA described above.

### RNA-seq library preparation and sequencing

Plants underwent one of four treatments: control (mock, nightly dark environment with MgCl_2_ treatment), RL (nightly red light treatment with MgCl_2_ treatment), DC3000 (nightly dark environment with DC3000 inoculation), and RL + DC3000 (nightly red light treatment with DC3000 inoculation). Leaves were collected from the plants at 5:00 AM the next day after treatment and immediately frozen in liquid nitrogen for further RNA exaction. Three biological replicates were sequenced for each treatment and at least three plants were pooled for each biological replicate. The enrichment of mRNA, fragment interruption, addition of adapters, size selection, PCR amplification, and RNA-seq were performed by staff at Zhejiang Tianke (Hangzhou, China). Poly (A) mRNA was isolated using oligo dT beads and then cleaved into short fragments. A single-end RNA-seq library was prepared for 12 samples from four different treatments, and sequenced on the Illumina HiSeq™ 2000 platform.

To identify genes regulated by RL and DC3000 in tomato leaves, we selected the genes whose expression was altered by treatments of RL, DC3000 and RL + DC3000 compared with the mock control. A combination of FDR (false discovery rate) ≤ 0.05 and the absolute value of log2 Ratio ≥ 2 were used as the threshold to judge the significance of gene expression difference [29].

### Analysis of Illumina sequencing results

The raw reads generated from the sequencing machines were cleaned by discarding the adaptor sequences and low-quality reads and filtering the reads with an unknown nucleotide percentage greater than 5%. The mapping of clean reads (from the single-end RNA-seq library) onto the tomato genome (The International Tomato Annotation Group *Solanum lycopersicum* protein reference version 2.0 reference) was conducted with Bowtie using the default parameters. The sequences from the Illumina sequencing were deposited in the NCBI Sequence Read Archive database (Accession GSE64087, http://www.ncbi.nlm.nih.gov/geo/query/acc.cgi?acc=GSE64087). Gene expression was quantified as the total number of reads for each sample that uniquely aligned to the reference. The functional annotation software Blast2go Program [30] was used to assign gene ontology (GO) terms. WEGO was utilised to classify GO function [31].

### Verification of RNA-seq results by real-time PCR (qRT-PCR)

To synthesis cDNA, total RNA from diluted stocks of the same RNA that was subjected to RNA-seq was used in each reverse transcription reaction using the ReverTra Ace qRT-PCR Kit (Toyobo, Japan). qRT-PCR was performed using SYBR-Green chemistry and the iCycler iQ™ Real-Time PCR Detection System (Bio-Rad, Hercules, CA, USA) [32]. The primers used to amplify the targeted genes were designed using Primer Premier 5.0 (Additional file 3: Table S2). Melting curve analysis of the PCR products was conducted at the end of each PCR cycle to verify the amplicon specificity. The mRNA expression levels of the target genes were normalized relative to the expression of the housekeeping gene *actin2* to minimise sample variation. All qRT-PCR reactions were repeated with three independent biological replicates and two technical replicates. The data were analysed based on the method of Livak and Schmittgen [33].

### Statistical analysis

The statistical analysis for the data except RNA-seq data was performed by analysis of variance (ANOVA) by using Duncan’s multiple range test (*p* < 0.05).

## Results

### Diurnal changes in the resistance to DC3000 pathogens

**Figure 1:**
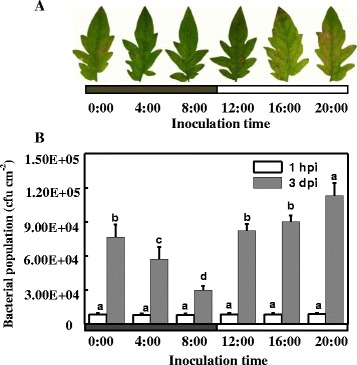
**Effects of DC3000 inoculation time on disease development. (A)** Photographs were taken 6 days after DC3000 inoculation. **(B)** Bacterial populations were measured at 1 hour and 3 days post-inoculation (OD = 0.1). The data represent mean values (± SD) of colony forming units (CFU) per square centimeter from five biological replicates, each replicate consisting of three leaf discs. Means denoted by the same letter did not significantly differ at *p* < 0.05 according to Duncan’s multiple range test. Initial bacterial numbers (1hpi) was quantified to ensure the uniformity of inoculation of DC3000. Black and white boxes correspond to dark and light periods, respectively, during a normal growth chamber day. The experiment was repeated twice with similar results.

To investigate the diurnal changes in plant defence, we inoculated tomato plants every 4 h with DC3000 pathogens and examined the changes in resistance. As shown in Figure 1, there were significant differences in the pathogen population in the leaves at 3 day post-inoculation (dpi). The leaves that were inoculated with DC3000 at 8:00 AM had the lowest pathogen population, followed by those inoculated at 4:00 AM. In comparison, the leaves that were inoculated with DC3000 at 8:00 PM had the highest pathogen population. These results clearly demonstrate that disease resistance in tomato leaves is remarkably influenced by the time of DC3000 inoculation and that the resistance of plants to DC3000 is lowest before midnight.

### Nightly red light treatment enhances resistance to DC3000

**Figure 2:**
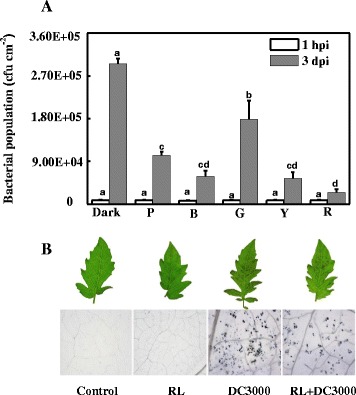
**Effects of nightly light treatment on the tomato defence resistance against DC3000. (A)** Bacterial populations were measured at 1 hour and 3 days post-inoculation (OD = 0.1). Dark, no nightly light treatment; P, night lighting with purple light; B, night lighting with blue light; G, night lighting with green light; Y, night lighting with yellow light; R, night lighting with red light. The data represent mean values (± SD) of colony forming units (CFU) per square centimeter from five biological replicates, each replicate consisting of three leaf discs. Means denoted by the same letter did not significantly differ at *p* < 0.05 according to Duncan’s multiple range test. Initial bacterial numbers (1 hpi) was quantified to ensure the uniformity of inoculation of DC3000. **(B)** Photographs were taken 6 d after DC3000 inoculation; Microscopic lesions of representative tomato leaf samples at 3 days after DC3000 dipping (40-fold magnification). The experiment was repeated twice with similar results.

To determine if light plays a role in the defence response, tomato plants inoculated with DC3000 were exposed at night to lights of different wavelengths supplied by LED lamps. Compared to the dark night control, purple (P), blue (B), green (G), yellow (Y) and red (R) light all suppressed pathogen growth, especially the red light treatment which showed the lowest amount of colony-forming units, while those of green light showed a smaller effect on immunity (Figure 2A). Trypan blue staining demonstrated that nightly red light treatment alleviated pathogen-induced cell death (Figure 2B).

**Figure 3:**
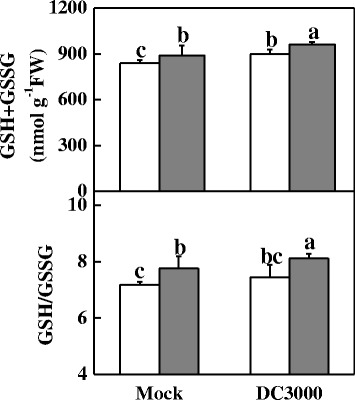
**Effects of DC3000 pathogens and red light alone or in combination on the glutathione content, and GSH/GSSG ratio in leaves of tomato plants at 19 h post-inoculation.** The plants were kept in the dark (open column) or under red light (grey column) conditions at night without (Mock) or with the immediate inoculation of DC3000. Data are the mean ± SD of five biological replicates. Means denoted by the same letter did not differ significantly at *p* < 0.05 according to Duncan’s multiple range test. The experiments were repeated twice with similar results.

To further determine the effects of nightly supplemental red light on plant resistance against DC3000 infection, we measured the light-saturated rate of CO_2_ assimilation (*A*_*sat*_), maximum quantum yield of PSII (*Fv/Fm*), chlorophyll content, and membrane damage extent. Compared with mock treatment, DC3000 infection significantly decreased *A*_*sat*_, and chlorophyll content, irrespective of light condition at night. Significantly, plants for nightly red light treatment showed higher *A*_*sat*_ and chlorophyll content than those for dark control after DC3000 infection. Also, nightly red light treatment significantly alleviated DC3000-induced reductions in *Fv/Fm* and membrane damage as indicated by the decreased relative electrolyte leakage (EL) in the leaves (Additional file 4: Figure S2). *Fv/Fm* and EL for RL + DC3000 treated plants were 4.8% higher and 65.6% lower than those for DC3000 treated plants, respectively. Furthermore, RL and DC3000 treatment alone or in combination increased cellular glutathione accumulation significantly. Nightly RL also increased the ratio of GSH to GSSG in the presence of DC3000 (RL + DC3000) compared to DC3000 treatment alone (Figure 3).

**Figure 4:**
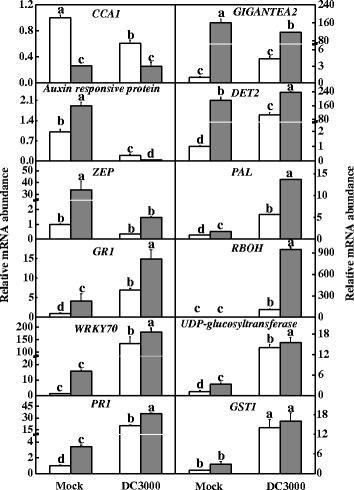
**Effects of the DC3000 pathogen and red light alone or in combination on the transcription of several defence-related genes in leaves of tomato plants at 19 h post-inoculation.** The plants were kept in the dark (open column) or under red light (grey column) conditions at night without (Mock) or with the immediate inoculation of DC3000. Data are the mean ± SD of three biological replicates with two technical replicates. Means denoted by the same letter did not differ significantly at *p* < 0.05 according to Duncan’s multiple range test. The experiments were repeated twice with similar results.

**Figure 5:**
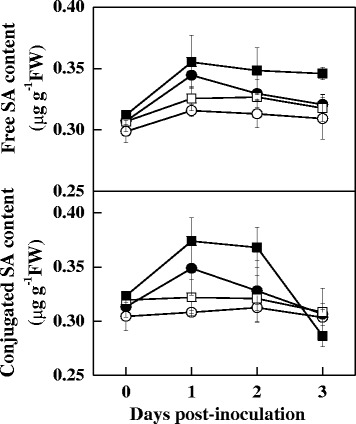
**Time-course of the free and conjugated SA contents in tomato leaves as influenced by DC3000 infection and red light.** The plants were kept in the dark (circle symbols) or under red light (squares symbols) conditions at night without (open symbols) or with the immediate inoculation of DC3000 (closed symbols). Data are the mean ± SD of five biological replicates. Means denoted by the same letter did not differ significantly at *p* < 0.05 according to Duncan’s multiple range test.

To examine the effect of red light on gene transcription, RNA was isolated from leaves at 5:00 AM, 9 h after red light exposure from 8:00 PM for the subsequent qRT-PCR analysis. The qRT-PCR analysis demonstrated that both RL and DC3000 treatments alone or in combination induced the transcription of a subset of genes including *PR1*, *glutathione reductase 1* (*GR1)*, *PAL*, *glutathione S-transferases* (*GST1)*, *GIGANTEA2* (*GI2*), *UDP-glucuronosyltransferase* (*UGT*), and *WRKY70*, which are involved in the defence response and the light response; the induction was most significant in the RL + DC3000 treatment (Figure 4). Time-course analysis for the transcription of *PR1, GR1, PAL* and *ICS* also showed that these genes were largely upregulated by red light within 3 dpi (Additional file 5: Figure S3). Meanwhile, RL and DC3000 treatment alone or in combination resulted in increased free and conjugated SA accumulation in leaves; this accumulation was the most significant in the RL + DC3000 treatment (Figure 5). These results suggest that nightly RL treatment improved resistance to DC3000 in tomato plants and this response was associated with increased SA signalling pathway activity.

### Transcriptome profiling by RNA-seq

**Figure 6:**
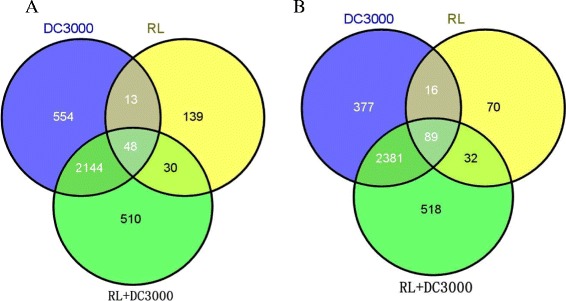
**Expression of genes in RL, DC3000, and RL + DC3000.** Venn diagrams of differential expressed genes in the three different treatments. **(A)** Up-regulated genes, **(B)** Down-regulated genes. RL indicates nightly red light with MgCl_2_ treatment; DC3000 indicates a nightly dark environment with DC3000 inoculation; RL + DC3000 indicates DC3000 inoculation with nightly red light treatment.

To examine how nightly red light treatment enhances the resistance to DC3000, we performed RNA-seq analysis using tomato leaves treated with and without the DC3000 pathogen and nightly RL. RNA-seq was performed on an Illumina HiSeq™ 2000 sequencer, which generated a total of 165,226,005 1*50 reads from all samples. Each sample’s reads were aligned to the *Solanum lycopersicum* reference genome (Table 1). All treatments were compared to the mock control, and > 2.0 log_2_ fold-change and a *q*-value less than 0.05 were regarded as a significant difference. Compared with the control (mock), nightly red light treatment (RL), DC3000 inoculation (DC3000), and RL + DC3000 (nightly red light treatment with DC3000 inoculation) differentially changed the transcription of a total of 437 (230 up-regulated, 207 down-regulated), 5,622 (2,759 up-regulated and 2,863 down-regulated), and 5,752 (2,732 up-regulated and 3,020 down-regulated) genes, respectively (Figure 6; Additional file 6: Table S3; Additional file 7: Table S4; Additional file 8: Table S5; Additional file 9: Table S6; Additional file 10: Table S7). Among them, a total of 137 genes (48 up-regulated and 89 down-regulated genes) were commonly regulated by the three different treatments. Among the up-regulated genes, a high number of genes overlapped between RL and DC3000 (61 genes). In total, 78 genes were induced by both RL and RL + DC3000, whereas 2,192 genes were induced by both DC3000 and RL + DC3000. A large overlap was also found among the down-regulated genes in distinct treatments. In the RL + DC3000 treatment, 178 genes were up-regulated, and 176 genes were down-regulated compared with DC3000 treatment (Figure 6). These results suggest that nightly RL had a significant effect on the transcription of a subset of genes. The results of the qRT-PCR analysis were very similar to those obtained by RNA-seq analysis across all treatments (r = 0.91; *p* < 0.0001, Additional file 11: Table S8 and Additional file 12: Figure S4), indicating that the changes in expression detected by RNA-seq were accurate.

**Table 1:** **Transcriptome alignment data and assembly statistics**

**Samples**	**Total number of sequenced reads**	**Total number of mapped reads**	**Total number of uniquely mapped reads**	**Average unique mapping (%)**
Control	13,516,400 ± 1,741,915	9,974,724 ± 1,292,810	9,684,205 ± 1,223,926	97.10% ± 0.44%
DC3000	13,576,548 ± 2,450,052	10,178,545 ± 1,785,204	9,667,734 ± 1,709,733	94.93% ± 0.15%
RL	13,430,307 ± 1,119,471	9,847,503 ± 816,537	9,496,471 ± 780,062	96.43% ± 0.06%
RL + DC3000	14,552,081 ± 1,064,416	10,982,567 ± 830,359	10,438,225 ± 733,054	95.07% ± 0.51%
Total	165,226,005	122,950,014	117,859,905	95.88%

GO analysis was used to determine the functions of these up-regulated and down-regulated genes (Additional file 13: Figure S5). In the cellular component categories, cell, organelle part, and macromolecular complex were the most abundant GO terms induced by either DC3000 or RL. Within the biological process categories, a large number of DC3000- and RL-responsive genes belonged to the categories of biological regulation, cellular process, metabolic process, and response to stimulus. In terms of molecular function, DC3000 and RL both induced the transcription of a subset of genes with transcription regulator, catalytic, binding, or transporter activity.

### Transcriptome profiles of circadian rhythm- and photosynthesis-related genes

Circadian rhythm plays a role in pathogen defence [13,14,34,35]. To investigate the involvement of circadian rhythm-related genes in the RL and DC3000 responses, we compared the transcriptome profiles of the control, DC3000, RL, and RL + DC3000 treatments. The DC3000 treatment had little effect on the induction of circadian rhythm genes (Table 2). RL induced the transcription of a subset of genes involved in circadian rhythms, such as *GI2* (Solyc12g056650), *GI1* (Solyc04g071990)*, timing of CAB expression 1* (*TOC1*) / *pseudo-response regulator 1* (*PRR1*) (Solyc03g115770), *PRR3* (Solyc04g049680), *PRR5* (Solyc10g005030), *PRR7* (Solyc10g086000), *flavin-binding kelch domain F box protein* (*FKF1,* Solyc01g005300), and *early flowering 4* (*ELF4,* Solyc06g051680), in the presence or absence of the DC3000 pathogen. The transcription of these circadian rhythm-related genes was thus greater in nightly supplementary RL plants than in the dark control after DC3000 infection. Similarly, there were no significantly differences in the transcription of circadian rhythm-related genes between RL + DC3000 and RL treatment, suggesting that DC3000 had little effects on the circadian rhythm expression (Table 2).

**Table 2:** **Circadian rhythm and photosynthesis related genes expression in tomato leaves as influenced by RL and DC3000 alone or in combination treatment**

**Gene ID**	**DC3000**		**RL**		**RL + DC3000**		**(RL + DC3000)** **-DC3000**		**Description**
	**Log**_**2**_ **FC**	***q*** **-value**	**Log**_**2**_ **FC**	***q*** **-value**	**Log**_**2**_ **FC**	***q*** **-value**	**Log**_**2**_ **FC**	***q*** **-value**	
**Circadian rhythm**									
Solyc12g056650	2.49	3.24E-01	7.91	9.16E-09	7.44	4.71E-07	4.95	4.15E-06	GIGANTEA 2
Solyc04g071990	1.02	3.28E-01	4.66	1.47E-09	5.54	1.73E-17	4.52	8.34E-15	GIGANTEA 1
Solyc03g115770	1.41	1.74E-03	2.80	4.40E-16	2.50	1.11E-11	1.09	1.68E-03	Timing of CAB expression 1/ Pseudo-response regulator 1
Solyc04g049680	−1.68	1.84E-02	4.36	9.75E-75	3.68	1.09E-39	5.36	3.07E-43	Pseudo-response regulator 3
Solyc10g005030	−0.09	5.18E-01	4.63	4.12E-03	4.73	6.83E-04	4.82	5.61E-03	Pseudo-response regulator 5
Solyc10g086000	0.14	4.99E-01	3.38	1.08E-06	3.73	3.03E-09	3.59	1.12E-07	Pseudo-response regulator 7
Solyc01g005300	1.45	1.70E-01	3.34	2.88E-04	3.74	2.45E-06	2.29	2.06E-03	Flavin-binding kelch domain F box protein
Solyc06g051680	0.90	3.05E-01	4.33	1.02E-11	3.70	2.30E-07	2.80	6.49E-05	Early flowering 4
Solyc10g005070	−0.90	3.02E-05	−1.87	6.50E-14	−4.66	1.00E-30	−3.76	1.45E-13	Circadian clock associated 11
Solyc10g005080	−1.43	3.27E-15	−2.24	8.66E-29	−3.72	2.07E-44	−2.29	9.68E-10	Late elongated hypocotyl
Solyc02g036370	−0.48	8.29E-10	−1.80	1.36E-82	−3.04	3.27E-141	−2.56	3.46E-79	Late elongated hypocotyl
**Photosynthesis**									
Solyc05g010240	−1.69	3.60E-01	2.63	1.71E-01	−2.49	3.03E-01	−0.80	1.00E + 00	Rubisco large subunit-binding protein subunit beta
Solyc12g044280	−5.58	0.00E + 00	2.14	0.00E + 00	−5.51	0.00E + 00	0.07	1.00E + 00	Photosystem I reaction center subunit VI
Solyc02g071030	−4.98	2.14E-230	4.09	0.00E + 00	−3.38	2.61E-186	1.60	2.54E-07	Chlorophyll a/b binding protein

Shown are the log_2_ fold-changes values (log_2_FC) and *q*-value (≤0.05) for genes expression by DC3000, RL, RL + DC3000, and (RL + DC3000)-DC3000 (comparison between RL + DC3000 and DC3000).

Similar to the transcriptome profiles of circadian rhythm-related genes, the DC3000 and RL treatments induced quite different transcriptome profiles of photosynthesis-related genes. The transcription of a number of genes involved in photosynthesis was down-regulated by the DC3000 treatment. In the absence of the DC3000 pathogen, RL induced the transcription of genes encoding chlorophyll a/b-binding protein (Solyc02g071030), photosystem I reaction centre subunit VI (Solyc12g044280), and RuBisCO large subunit-binding protein subunit beta (Solyc05g010240) by more than 4-fold. However, the induction of these gene transcriptions by RL was largely eliminated in the presence of the DC3000 pathogen (RL + DC3000 treatment). Finally, RL + DC3000 treatment had higher gene transcription of *chlorophyll a/b-binding protein* as compared with those in DC3000 treatment (Table 2).

### Transcriptome profiles of hormone metabolism and signalling-related genes

Multiple hormonal pathways are often modulated by light to mediate developmental changes. RL and DC3000 treatment alone or in combination all repressed the transcription for nearly all of the genes in the auxin-mediated signalling pathways (Table 3). These genes include *auxin-induced SAUR* and *SAUR*--like protein (Solyc01g110790, Solyc03g033590, Solyc04g053010, and Solyc11g011670) and *auxin-responsive protein IAAs* (Solyc06g084070, Solyc06g008590). However, *indole-3-acetic acid-amido synthetase* GH3-8 (Solyc02g092820) was significantly induced in the presence of the DC3000 pathogen. In addition, RL + DC3000 treatment showed lower transcriptions of auxin responsive protein (Solyc06g008590) and *indole-3-acetic acid-amido synthetase GH3.8* (Solyc02g092820) compared to DC3000 treatment (Table 3).

**Table 3:** **Hormone metabolism and signalling-related gene expression in tomato leaves as influenced by RL and DC3000 alone or in combination treatment**

**Gene ID**	**DC3000**		**RL**		**RL + DC3000**		**(RL + DC3000) -DC3000**		**Description**
	**Log**_**2**_ **FC**	***q*** **-value**	**Log**_**2**_ **FC**	***q*** **-value**	**Log**_**2**_ **FC**	***q*** **-value**	**Log**_**2**_ **FC**	***q*** **-value**	
**Auxin**									
Solyc01g110790	−4.72	2.23E-04	−2.34	2.20E-02	−3.56	6.28E-04	n.d.	n.d.	Auxin-induced SAUR-like protein
Solyc03g033590	−2.61	1.58E-03	−4.01	4.66E-04	−3.27	4.02E-04	−0.66	1.00E + 00	Auxin-induced SAUR-like protein
Solyc04g053010	−5.16	9.47E-04	−3.17	1.58E-02	−4.38	1.26E-03	n.d.	n.d.	Auxin-induced protein 6B
Solyc11g011670	−2.99	4.94E-02	−3.5	1.47E-01	−3.29	4.05E-02	n.d.	n.d.	Auxin-induced protein 6B
Solyc06g084070	−3.18	4.93E-02	−3.67	1.55E-01	−3.2	4.81E-02	n.d.	n.d.	Auxin responsive protein IAA2
Solyc06g008590	−4.57	5.07E-04	−2.04	6.10E-02	−5.93	3.48E-04	−1.36	1.00E + 00	Auxin responsive protein IAA10
Solyc02g092820	7.11	2.04E-18	0.48	1.00E + 00	5.34	1.48E-06	−1.77	9.03E-07	Indole-3-acetic acid-amido synthetase GH3-8
**Salicylic acid**									
Solyc06g071030	−3.92	6.42E-04	0.24	1.00E + 00	−4.35	4.18E-04	−0.43	1.00E + 00	Isochorismate synthase
Solyc09g007910	2.85	0.00E + 00	−0.51	2.23E-04	2.18	1.59E-170	−0.67	6.95E-42	Phenylalanine ammonia-lyase
Solyc03g036480	6.34	3.62E-10	n.d.	n.d.	5.43	4.41E-06	−0.91	1.17E-01	Phenylalanine ammonia-lyase
**Jasmonate**									
Solyc03g122340	6.3	1.73E-125	1.01	4.20E-01	5.82	4.45E-92	−0.48	2.08E-05	Lipoxygenase
Solyc08g014000	8.06	6.83E-22	−0.39	1.00E + 00	6.33	2.88E-08	−1.73	1.69E-08	Lipoxygenase
Solyc04g079730	7.22	1.42E-27	0.71	1.00E + 00	6.17	9.23E-15	−1.05	7.37E-05	Allene oxide synthase
Solyc09g089540	8.01	0.00E + 00	0.81	1.99E-01	8.61	0.00E + 00	0.6	5.56E-103	Proteinase inhibitor I
Solyc09g089500	8.59	0.00E + 00	1.19	3.96E-01	8.77	0.00E + 00	0.18	1.21E-03	Proteinase inhibitor I
Solyc09g084440	1.12	2.21E-02	−2.42	3.13E-02	0.48	2.70E-01	−0.64	5.25E-01	Proteinase inhibitor I
Solyc03g020060	9.14	0.00E + 00	0.51	9.43E-01	9.1	0.00E + 00	−0.04	6.54E-01	Proteinase inhibitor II
Solyc00g145170	9.9	1.62E-77	1.44	9.98E-01	9.8	1.07E-73	−0.11	7.35E-01	Proteinase inhibitor II
Solyc06g063390	−0.44	1.70E-01	−2.2	1.82E-05	−1.24	2.66E-03	−0.8	3.00E-01	Wound-induced basic protein
Solyc12g009220	8.27	2.95E-188	1.66	2.36E-01	9	9.73E-289	0.73	1.28E-31	Jasmonate ZIM-domain protein 1
**Ethylene**									
Solyc08g081540	3.74	4.76E-04	−0.4	1.00E + 00	2.17	1.00E-01	−1.57	1.55E-01	1-aminocyclopropane-1-carboxylate synthase
Solyc12g005940	8.86	0.00E + 00	1.36	6.10E-02	9.17	0.00E + 00	0.32	1.24E-22	1-aminocyclopropane-1-carboxylate oxidase
Solyc09g065310	4.12	5.66E-03	n.d.	n.d.	n.d.	n.d.	−3.99	3.99E-02	Ethylene receptor 2
Solyc08g007230	5.25	1.86E-05	n.d.	n.d.	n.d.	n.d.	−1.96	3.09E-02	Ethylene responsive transcription factor 1a
**Brassinosteroid**									
Solyc01g009810	5.36	2.00E-17	0.08	1.00E + 00	6.39	1.97E-34	1.03	1.27E-05	LRR receptor-like serine/threonine-protein kinase FEI 1
Solyc10g086500	4.15	1.52E-59	3.53	1.52E-37	3.66	1.08E-39	−0.49	3.19E-03	Steroid 5-alpha-reductase DET2
Solyc09g092520	−2.64	4.11E-15	−2.37	8.20E-14	−2.21	1.24E-12	0.43	8.66E-01	Brassinosteroid-regulated protein xyloglucan endotransglycosylase
Solyc02g086180	−4.14	7.44E-289	2.15	0.00E + 00	−4.21	9.70E-294	−0.07	1.00E + 00	Sterol C-5 desaturase
Solyc06g074090	−3.04	1.54E-08	2.27	2.37E-28	−3.57	1.10E-09	−0.53	1.00E + 00	Sterol reductase
Solyc04g074450	−2.8	1.91E-09	−3.28	8.89E-11	−2.79	1.84E-09	0.02	1.00E + 00	Phi-1 protein
**Other hormone pathway**									
Solyc02g090890	−2.07	7.02E-03	3.16	3.59E-26	0.64	1.48E-01	2.71	8.83E-04	Zeaxanthin epoxidase chloroplastic
Solyc04g071960	−3.67	4.30E-05	1.36	9.83E-04	−3.79	3.29E-05	−0.12	1.00E + 00	Xanthoxin dehydrogenase
Solyc08g081370	1.49	2.67E-25	−0.65	8.60E-03	0.32	6.35E-02	−1.17	4.46E-16	RING-H2 finger protein
Solyc01g109170	3.28	1.21E-292	−1.16	6.63E-09	2.7	1.34E-164	−0.58	5.20E-22	Cold acclimation protein COR413-like
Solyc03g095780	2.35	1.23E-30	−1.13	1.15E-02	0.48	8.11E-02	−1.87	3.24E-21	Abscisic acid receptor PYL6
Solyc06g049050	−5.18	0.00E + 00	−1.45	0.00E + 00	−7.78	0.00E + 00	−2.6	1.07E-21	Expansin
Solyc01g090460	−2.66	1.95E-09	−1.89	2.72E-06	−4.2	2.55E-13	−1.54	3.56E-01	Homeobox-leucine zipper protein
Solyc12g056650	2.49	3.24E-01	7.91	9.16E-09	7.44	4.71E-07	4.95	4.15E-06	GIGANTEA2
Solyc04g050930	−4.03	1.49E-12	0.53	1.45E-01	−4.32	4.32E-13	−0.29	1.00E + 00	Violaxanthin de-epoxidase

Shown are the log_2_ fold-changes values (log_2_FC) and *q*-value (≤0.05) for genes expression by DC3000, RL, RL + DC3000, and (RL + DC3000)-DC3000 (comparison between RL + DC3000 and DC3000). The n.d. means not detectable.

Infection by DC3000 in tomato leaves up-regulated the transcription of the SA biosynthesis gene *PAL* (Solyc09g007910, Solyc03g036480) in the phenylpropanoid pathway but down-regulated another SA biosynthesis pathway gene *isochorismate synthase* (*ICS*, Solyc06g071030). By contrast, RL treatment slightly up-regulated the transcription of *ICS* but down-regulated that of *PAL* (Table 3). JA biosynthesis genes including those encoding lipoxygenases (*LOXs*, Solyc03g122340, 78.6-fold; Solyc08g014000, 266.9-fold) and allene oxide synthase (*AOS*, Solyc04g079730, 149.1-fold) as well as *PI I* (Solyc09g089540, 257.8-fold; Solyc09g089500, 385.3-fold) and *PI II*, (Solyc03g020060, 564.2-fold; Solyc00g145170, 955.4-fold) were induced by DC3000 infection. However, transcriptome analysis revealed reductions in the JA-regulated gene *PI I* (Solyc09g084440), and *wound-induced basic protein* (Solyc06g063390) in the RL treatment. Notably, the *jasmonate ZIM domain-containing protein 1* (Solyc12g009220, 3.15-fold) was up-regulated by RL treatment. However, transcription of these genes in the RL + DC3000 was similar to that in the DC3000 treatment (Table 3). The ethylene biosynthesis genes *ACC synthase* (*ACS*, Solyc08g081540, 13.38-fold) and *ACC oxidase* (*ACO*, Solyc12g005940, 463.4-fold) were activated concomitant with the induction of the *ethylene receptor* (*ETR,* Solyc09g065310, 17.36-fold) and *ethylene-responsive transcription factor 1a* (*ERF*, Solyc08g007230, 38.10-fold) upon infection with DC3000. However, the transcription of ethylene-related genes was primarily down-regulated by RL after DC3000 infection (Table 3).

Exposure to DC3000 resulted in the down-regulation of most genes involved in BRs metabolism and signalling with the exception of the BRs biosynthetic genes *steroid 5-alpha-reductase DET2* (Solyc10g086500) and signal transduction gene *LRR receptor-like serine/threonine-protein kinase FEI 1* (Solyc01g009810). In comparison, RL treatment induced a subset of genes involved in BRs signalling cascades while depressed a number of other genes (Table 3).

Transcription of *zeaxanthin epoxidase chloroplastic* (*ZEP*, Solyc02g090890), *xanthoxin dehydrogenase* (*XDH*, Solyc04g071960), and *violaxanthin de-epoxidase* (*VDE*, Solyc04g050930) were down-regulated by DC3000 infection, while *ZEP* and *XDH* were up-regulated by RL treatment. By contrast, *RING-H2 finger protein* (Solyc08g081370), *cold acclimation protein COR413*-like (Solyc01g109170), and *abscisic acid receptor PYL6* (Solyc03g095780) were up-regulated by DC3000 infection but down-regulated by RL treatment. Meanwhile, DC3000 and RL treatment both resulted in the up-regulation of the transcription for *GI2* (Solyc12g056650) but the down-regulation of *expansin* (Solyc06g049050) and *homeobox-leucine zipper protein* (*HD-ZIP*, Solyc01g090460). Compared with DC3000 treatment alone, RL combined with DC3000 treatment upregulated the transcription of *ZEP* and *GI2* while decreased the transcription of a number of other genes (Table 3).

### Transcriptome profiles of induced calcium signalling- and redox-related genes

Calcium and reactive oxygen species are crucial for the development of plant defences against abiotic and biotic stimuli. Here, we found that DC3000 treatment up-regulated the transcription of *calcium-dependent protein kinase* (*CDPK*, Solyc02g083850), *CAM* (Solyc06g053930), *calmodulin-like* protein (*CLP*, Solyc03g115930), and *calmodulin-binding protein* (*CBP*, Solyc07g040710) both in the presence and absence of RL. RL treatment alone had little effect on the transcription of most of these genes. Compared with DC3000, RL in presence of DC3000 infection upregulated the transcription of *CLP* (Solyc03g115930), *calcium-binding EF-hand family protein-like* (Solyc03g031630) (Table 4).

**Table 4:** **Calcium signalling- and redox-related genes expression in tomato leaves as influenced by RL and DC3000 alone or in combination treatment**

**Gene ID**	**DC3000**		**RL**		**RL + DC3000**		**(RL + DC3000) -DC3000**		**Description**
	**Log**_**2**_ **FC**	***q*** **-value**	**Log**_**2**_ **FC**	***q*** **-value**	**Log**_**2**_ **FC**	***q*** **-value**	**Log**_**2**_ **FC**	***q*** **-value**	
**Calcium**									
Solyc02g083850	5.79	4.32E-39	0.4	1.00E + 00	5.29	5.57E-28	−0.50	3.51E-02	Calcium-dependent protein kinase
Solyc06g053930	4.99	6.04E-04	0.14	1.00E + 00	5.91	1.68E-06	0.92	3.10E-01	Calmodulin
Solyc03g115930	3.65	3.04E-15	0.17	1.00E + 00	5.87	4.54E-80	2.22	6.46E-38	Calmodulin-like protein
Solyc07g040710	7.46	4.88E-14	0.78	1.00E + 00	7.04	3.39E-11	−0.42	5.09E-01	Calmodulin-binding protein
Solyc03g031630	n.d.	n.d.	n.d.	n.d.	7.52	2.70E-20	6.30	1.66E-19	Calcium-binding EF-hand family protein-like
**Redox**									
Solyc09g011580	2	1.90E-06	2.09	8.49E-07	3.13	3.56E-20	1.13	4.86E-05	Glutathione S-transferase-like protein
Solyc05g006860	4.07	4.33E-33	−0.11	1.00E + 00	6.12	1.21E-149	2.05	7.81E-65	Thioredoxin H
Solyc08g083360	−3.04	2.15E-269	0.25	2.48E-06	−4.38	0.00E + 00	−1.34	3.32E-11	Ferredoxin
Solyc03g005190	−3.4	1.42E-119	0.1	5.72E-01	−3.05	1.53E-109	0.35	5.33E-01	Ferredoxin
Solyc12g013810	−3.31	1.15E-272	0.09	3.19E-01	−3.28	3.60E-272	0.04	1.00E + 00	Thioredoxin
Solyc07g063190	−3.48	0.00E + 00	0.27	4.05E-15	−3.36	0.00E + 00	0.12	8.17E-01	Thioredoxin
Solyc08g062970	−3.38	5.60E-05	1.05	2.70E-02	−0.1	4.77E-01	3.28	1.14E-03	Glutaredoxin
Solyc04g011880	−2.85	2.95E-16	−0.06	1.00E + 00	−5.39	1.17E-23	−2.54	3.13E-02	Glutaredoxin
Solyc01g099620	n.d.	n.d.	n.d.	n.d.	6.05	1.10E-08	3.16	1.37E-05	Respiratory burst oxidase-like protein

Shown are the log_2_fold-changes values (log_2_FC) and *q*-value (≤0.05) for genes expression by DC3000, RL, RL + DC3000, and (RL + DC3000)-DC3000 (comparison between RL + DC3000 and DC3000). The n.d. means not detectable.

DC3000 treatment up-regulated the transcription of *glutathione S-transferase-like proteins* (Solyc09g011580) and *thioredoxin H* (Solyc05g006860) but down-regulated the transcription of *thioredoxin* (Solyc12g013810, Solyc07g063190), *ferredoxin* (Solyc08g083360, Solyc03g005190), and *glutaredoxin* (Solyc08g062970, Solyc04g011880). Interestingly, RL induced a general increase in the transcription of some of these genes. Importantly, there was a significant increase in the transcription for most of the genes involved in redox homeostasis. For example, the transcript for *respiratory burst oxidase-like protein* (Solyc01g099620) was increased by 8.92-fold in the RL + DC3000 treatment compared to DC3000 (Table 4).

### Transcriptome profiles of transcription factors, post-transcription, and defence-related genes

While DC3000 treatment induced the transcription for *WRKY70* (Solyc09g015770), *WRKY60* (Solyc08g067340), *WRKY53* (Solyc01g095630), and *WRKY18* (Solyc08g067360), it suppressed the transcription of other transcription factors (TFs) such as *NAC domain class TF* (Solyc02g093420). RL treatment alone had negligible effects on the transcription of most TFs or even down-regulated the transcription of a *NAC domain class transcription factor* (Solyc02g093420), a *MADS-box transcription factor* (Solyc02g071730), a *bZIP transcription factor* (Solyc02g073580), and a *MYB transcription factor* (Solyc10g005070). Interestingly, RL + DC3000 treatment induced a significant increase in the transcription of *WRKY70*, *WRKY60*, *WRKY53*, *WRKY18*, *NAC* domain class *TF*, *BHLH TF* (Solyc02g063430), and *MADS*-box *TF* (Solyc02g071730) in the leaves challenged with DC3000 but further suppressed the transcription of other TFs compared with DC3000 (Table 5).

**Table 5:** **Transcription factors, post-transcription- and defence-related genes expression in tomato leaves as influenced by RL and DC3000 alone or in combination treatment**

**Gene ID**	**DC3000**		**RL**		**RL + DC3000**		**(RL + DC3000) -DC3000**		**Description**
	**Log**_**2**_ **FC**	***q*** **-value**	**Log**_**2**_ **FC**	***q*** **-value**	**Log**_**2**_ **FC**	***q*** **-value**	**Log**_**2**_ **FC**	***q*** **-value**	
**Transcription factors**									
Solyc09g015770	3.96	3.47E-36	0.87	3.61E-01	5.08	1.07E-86	1.12	1.03E-15	WRKY70
Solyc08g067340	6.01	3.98E-11	0.71	1.00E + 00	8.17	2.77E-40	2.16	5.32E-21	WRKY60
Solyc01g095630	2.14	2.31E-13	0.78	1.35E-01	3.22	8.19E-42	1.08	1.85E-09	WRKY53
Solyc08g067360	6.58	1.49E-08	0.13	1.00E + 00	9.00	3.21E-33	2.42	1.17E-21	WRKY 18
Solyc02g073580	−1.80	6.62E-20	−2.88	1.84E-36	−4.48	2.09E-47	−2.68	1.04E-08	BZIP transcription factor
Solyc10g005070	−0.90	3.02E-05	−1.87	6.50E-14	−4.66	1.00E-30	−3.76	1.45E-13	MYB transcription factor (Fragment)
Solyc02g093420	−2.25	3.39E-02	−1.73	2.72E-01	−0.01	5.20E-01	2.24	1.79E-01	NAC domain class transcription factor
Solyc02g071730	2.77	6.80E-02	−1.81	1.00E + 00	4.92	5.74E-06	2.15	1.08E-02	MADS-box transcription factor AGAMOUS
Solyc02g063430	−0.74	4.20E-01	0.37	1.00E + 00	3.74	4.33E-06	4.48	1.86E-05	BHLH transcription factor
**Post- transcription and defense genes**									
Solyc10g055790	6.33	3.84E-10	5.63	2.09E-06	5.10	4.71E-05	−1.23	2.39E-02	Chitinase 2
Solyc08g082670	−3.65	3.61E-03	2.22	2.29E-06	−1.01	1.60E-01	2.64	3.06E-01	Cellulose synthase
Solyc12g057070	3.21	1.39E-05	3.12	9.28E-05	4.50	1.04E-14	1.29	1.26E-03	UDP-glucuronosyltransferase
Solyc05g053400	−2.49	6.69E-02	3.87	1.89E-17	−1.89	1.12E-01	0.60	1.00E + 00	Glucosyltransferase
Solyc05g053120	−2.64	1.11E-01	4.20	1.68E-15	−2.07	1.53E-01	0.57	1.00E + 00	Glucosyltransferase
Solyc02g08735	6.26	n.d.	1.36	7.02E-04	7.03	4.84E-01	0.77	n.d.	Glycosyltransferase
Solyc03g043860	n.d.	n.d.	2.83	1.12E-02	0.21	n.d.	n.d.	n.d.	Nudix hydrolase 1
Solyc02g014250	n.d.	2.82E-01	4.30	4.49E-02	n.d.	n.d.	n.d.	n.d.	Blight resistance protein
Solyc07g009260	1.06	0.00E + 00	2.48	1.42E-45	n.d.	0.00E + 00	n.d.	1.41E-262	Defensin-like protein
Solyc08g074630	7.31	7.09E-18	2.54	8.12E-01	6.36	1.57E-10	−0.95	1.06E-01	Polyphenol oxidase
Solyc07g040690	2.58	7.82E-07	−0.49	1.00E-02	2.09	5.08E-29	−0.49	2.09E-11	Regulatory protein NPR1
Solyc00g272810	3.21	5.91E-03	2.30	1.00E + 00	5.13	1.59E-01	1.92	4.38E-01	N-acetyltransferase
Solyc04g082460	2.27	0.00E + 00	0.02	1.48E-54	1.25	0.00E + 00	−1.02	4.51E-01	Catalase
Solyc02g082760	−3.94	3.09E-272	−0.32	8.21E-01	−4.07	2.68E-256	−0.13	5.58E-01	Catalase
Solyc12g094620	−2.73	5.37E-202	0.05	9.09E-03	−2.57	8.06E-219	0.16	2.12E-01	Catalase
Solyc01g100630	−3.95	3.84E-10	−0.21	2.09E-06	−4.53	4.71E-05	−0.58	2.39E-02	Catalase

Shown are the log_2_ fold-changes values (log_2_FC) and *q*-value (≤0.05) for genes expression by DC3000, RL, RL + DC3000, and (RL + DC3000)-DC3000 (comparison between RL + DC3000 and DC3000). The n.d. means not detectable.

DC3000 treatment induced the transcription of *chitinase 2* (Solyc10g055790) and *UGT* (Solyc12g057070) but down-regulated the transcription of *cellulose synthase* (Solyc08g082670) and *GTs* (Solyc05g053400, Solyc05g053120). In comparison, RL treatment alone induced these genes compared with their dark control. Meanwhile, DC3000 treatment induced several genes involved in defence, such as *PPO* (Solyc08g074630) and regulatory protein *NPR1* (Solyc07g040690). Interestingly, RL also induced a subset of defence genes such as *NUDIX 1* (Solyc03g043860), blight resistance protein/putative disease resistance protein *RGA4* (Solyc02g014250), defensin-like protein (*DEFL,* Solyc07g009260), and *PPO* (Solyc08g074630). Finally, the RL + DC3000 treatment had higher transcription of a subset of genes such as *cellulose synthase* (Solyc08g082670), *UGT (*Solyc12g057070), and *N-acetyltransferase* (Solyc00g272810) compared to the DC3000 treatment (Table 5).

### Changes in the resistance against DC3000 pathogens after plants silenced with *NPR1* and *PI I/II*

**Figure 7:**
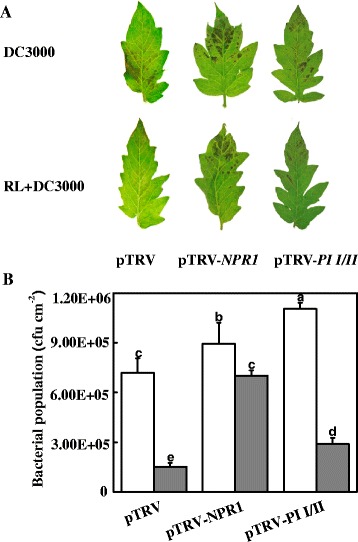
**DC3000 pathogen growth in leaves of tomato plants silenced with** ***NPR1*** **(pTRV-** ***NPR1*** **) and** ***PI I/II*** **(pTRV-** ***PI I/II*** **). (A)** Photographs were taken 6 days after DC3000 inoculation. **(B)** Bacterial populations were measured at 3 days post- inoculation (OD = 0.1). The data represent mean values (± SD) of colony forming units (CFU) per square centimeter from five biological replicates, each replicate consisting of three leaf discs. Means denoted by the same letter did not significantly differ at *p* < 0.05 according to Duncan’s multiple range test. White and Grey boxes correspond to dark and red light treatment, respectively, during a normal growth chamber day. The experiment was repeated twice with similar results.

To determine the role of SA and JA pathways in the RL-induced resistance against DC3000, we examined the changes in the resistance against DC3000 in tomato plants silenced with *NPR1* (pTRV-*NPR1*) and *PI I/II* (pTRV-*PI I/II*) with or without RL treatment. The results showed that *NPR1* and *PI I/II* gene expressions were down-regulated in pTRV-*NPR1* and pTRV-*PI I/II* plants (Additional file 14: Figure S6). pTRV-*NPR1* and pTRV-*PI I/II* plants both showed increased pathogen population as compared to the pTRV control plants. Significantly, RL treatment decreased the DC3000 population in the pTRV and pTRV-*PI I/II* plants by 78.9% and 73.8%. However, silencing of *NPR1* largely compromised RL-induced resistance against DC3000 pathogens and the DC3000 population decreased only 21.6% after the RL treatment (Figure7). Accordingly, SA signalling pathway played a role in RL-induced resistance against DC3000.

## Discussion

### Nightly red light-induced resistance to DC3000 is closely related to the diurnal rhythm

Our results demonstrated that there is a diurnal change in the resistance to DC3000 in tomato plants. The plants had the highest resistance to this pathogen at 8:00 AM and the greatest susceptibility at 8:00 PM (Figure 1); this result is consistent with previous observations [9,34]. Increased defence upon pathogen attack during the daytime has largely been attributed to the length of the light period following infection [9]. The diurnal changes in resistance to DC3000 are supposed to be associated with pathogen associated molecular pattern (PAMP)-triggered immune responses modulated by the circadian clock [34].

The exposure of plants to lights with different wavelengths differentially suppressed the disease occurrence; the effects of red light were the most significant (Figure 2). Red light induced the transcription of a series of genes involved in SA biosynthesis and defence (*PR1*, *RBOH*, *GST1*, *GR1*, *WRKY70*; Figure 4), which was followed by an increase in the GSH/GSSG ratio (Figure 3), suggesting that the SA dependent signaling pathway is involved in red light-enhanced resistance to DC3000 [36].

Phytochromes are required for SAR and to accommodate the supply of light energy required for the energetically costly increase in whole-plant resistance [9,37,38]. The circadian rhythm is mediated by phytochromes and cryptochromes [39]. Interestingly, PAMP-triggered immune responses against DC3000 are modulated by the circadian clock [13,34,35]. In our study, RNA-seq revealed that an array of genes (8 genes) involved in circadian rhythm was induced by red light treatment (Table 2). Among them, *ELF4* and *GIGANTEA* are involved in the phytochrome B (*PHYB*)-mediated signalling pathway, and *PHYB* is the main photoreceptor involved in perceiving and transducing the red-light signal [40]. The results suggest that the increased resistance to DC3000 by RL was partly attributable to the circadian rhythm–mediated modulation of PAMP-triggered immune [13,34,35]. In fact, numerous genes have been reported to be influenced by circadian rhythm [41] and some of them, like the genes of *GIGANTEA* (*GI*) and *N-acetyltransferase* (Solyc00g272810), have also been shown to regulate the tolerance or responses of plant to a series of biotic or abiotic stresses [42-44]. Furthermore, phytochrome signalling can modulate the SA-perceptive pathway and JA-dependent defence pathways to regulate plant defence [12,37]. For example, phyA, phyB, and phyC are required for resistance to *Magnaporthe grisea* in rice by regulating SA- and JA-dependent defence pathways [37]. Accordingly, the transcription involved in the circadian rhythm induced by RL may function as important mediators of RL-induced resistance to DC3000.

### Red light-induced resistance to DC3000 was correlated with enhancement of the SA pathway

The resistance of plants to pathogens is mainly regulated by three hormones: SA, JA, and ET. SA is an essential signalling molecule in the response to DC3000 attack. So far, two distinct enzymatic pathways, PAL-mediated phenylalanine pathway and ICS-mediated isochorismate pathway, for SA biosynthesis have been identified in plants. Though the ICS pathway is responsible for the majority of SA synthesis in pathogen-infected *Arabidopsis* and tobacco, our recent study also revealed that PAL pathway is responsible for SA biosynthesis [45]. In the present study, RNA-seq, qRT-PCR, and SA quantification revealed that DC3000 treatment increased SA biosynthesis and its signalling cascade probably in a PAL-dependent manner. By contrast, RL treatment failed to induce *PAL* transcription but slightly up-regulated the transcription of *ICS* (Table 3)*.* Also, inhibition of the catalase transcription (Solyc04g082460) suggests accumulation of SA in plant by RL under DC3000 infection (Table 5) [46]. It has been shown that SA synthesized is required for *PR1* gene expression and SAR defence responses [47]. Here, with the up-regulation of *ICS, PAL* and free SA content under red light exposure, the highest transcription of *PR1* and other SA downstream genes such as *NPR1, WRKY70*, *WRKY60*, and *WRKY18* were observed in the RL + DC3000 treatment (Table 5). Accordingly, it seems probable that the SA pathway play an important role in the RL-induced resistance to DC3000. In agreement with this, *NPR1* silencing partially abolished RL-induced resistance against DC3000 (Figure 7). This provided evidence that SA pathway was involved in the RL-induced resistance against DC3000 pathogens.

In addition to the induction of the SA pathway, DC3000 treatment also activated JA biosynthesis and signalling genes (Solyc03g122340 and Solyc04g079730) (Table 3). Induction of the JA pathway is believed to be a strategy by which the pathogen antagonises the host plant immune response [48]. In soybean, the expression levels of JA signalling and some JA biosynthesis genes are suppressed during fully established compatible cyst nematode infections [49]. Here, we found that transcription of JA-related genes in the RL + DC3000 treatment was not largely different from that of DC3000 treatment and silencing of *PI I/II* did not abolish RL-induced resistance (Table 3 and Figure 7), suggesting that JA signalling pathway was not mainly responsible for the RL-induced resistance against DC3000. ET has been observed for the induction of the defence response in many plants by up-regulating genes involved in ET production including *ACO* [50,51]. In the present study, transcription for ET-related genes was suppressed by RL treatment (Table 3), which may contribute to the activation of the SA pathway and possibly played a vital role in limiting *P. syringae* growth. In contrast to the up-regulation of the SA pathway, DC3000 treatment resulted in the down-regulation of a series of auxin- and BRs-responsive genes. While RL treatment down-regulated the transcription of auxin-responsive genes, it differentially up- or down-regulated BRs-responsive genes (*PHI-1*; *DET2*). Furthermore, the transcription of these genes in the DC3000 and RL + DC3000 treatments did not differ, suggesting that BRs and auxin were not responsible for the RL-induced resistance (Table 3). Although several ABA biosynthesis and downstream signalling genes were differentially altered by the DC3000 and RL treatments, it is difficult to determine their role in the RL-induced resistance to DC3000.

### Red light-induced redox homeostasis is involved in increased resistance against DC3000

As secondary signalling molecules, ROS and calcium are crucial for the development of plant defences against abiotic and biotic stimuli. Here, we found that RL up-regulated several important genes involved in cellular redox homeostasis (Table 4). Among them, *RBOH* is critical for the defence against DC3000, and the analogue *RbohF* mutant of *Arabidopsis* exhibits increased susceptibility to DC3000 [52]. RL induced the transcription of a subset of calmodulin-like proteins and calmodulin-binding proteins in the tomato plants in response to DC3000 challenge (Table 4). Calcium is a key factor in many adaptations and developmental processes in plants, and calcium signalling is crucial for the development of plant defences against abiotic and biotic stimuli. ROS signalling is integrated with calcium signalling in plants [53]. ROS-induced cellular redox changes have been previously reported in the regulation of NPR1, an essential regulator of SAR [54]. In our study, RL induced an increased GSH/GSSG ratio (Figure 3), which may contribute to the increased *PR1* transcription via *NPR1*.

We observed that several TFs, including *WRKY70*, *WRKY18*, *WRKY53*, and *WRKY60*, were highly up-regulated by RL in response to DC3000 challenge (Table 5). Acting downstream of *NPR1*, the overexpression of *WRKY70* results in the constitutive expression of SA-induced genes and increases resistance to SA-sensitive pathogens, while reducing resistance to JA-sensitive pathogens [55]. Accordingly, the up-regulation of these genes may play a role in RL-induced resistance to DC3000.

Glycosylation is not only involved in the regulation of cellular metabolism by altering the activity, solubility, and transport of aglycones such as plant hormones, secondary metabolites, and xenobiotics [56], but also in cell wall synthesis and plant defence [57]. The expression of the corresponding glycosyltransferase genes is essential in the hypersensitive response and nematode resistance as these genes could modify cellular redox homeostasis [58,59]. Many glycosyltransferase genes were up-regulated after DC3000 infection, and some glycosyltransferase genes were also induced by RL, suggesting a role for these genes in RL-induced resistance to DC3000 (Table 5). In addition to glycosyltransferases, cellulose synthase is another essential enzyme for the formation of the cell wall, which is the primary interface for plant-pathogen interactions [60-62]. In contrast to the down-regulation of cellulose synthase (Solyc08g082670, Solyc08g082660) upon DC3000 infection, RL exposure resulted in an up-regulation of the expression of cellulose synthase which may also contribute to the enhanced resistance to this pathogen (Table 5).

## Conclusions

Tomato plants exhibit diurnal changes in the susceptibility to DC3000 and are most susceptible in the evening. Nightly red light treatment significantly enhanced DC3000 resistance; this effect was accompanied with increased SA accumulation and defence-related gene transcription. RNA-seq analysis revealed that (1) red light induced a set of circadian rhythm-related genes involved in phytochrome- and SA-regulated resistance; (2) the biosynthesis and signalling pathways of multiple plant hormones (SA, auxin, JA, and ET) were co-ordinately regulated following DC3000 infection, with the most significant effect on the SA pathway, indicating that SA-mediated signalling pathways are involved in the red light-induced resistance to the pathogen; (3) a set of genes involved in redox homeostasis (*RBOH*, *GSTs*, *GTs*), calcium (calmodulin and calmodulin-binding protein), and defence (*PPO*, *NUDIX1*) as well as *TFs* (*WRKY18*, *WRKY 53*, *WRKY 60*, and *WRKY70*) were differentially induced at the transcriptional level by red light in response to pathogen challenge. Silencing of *NPR1*, an important gene involved in SA signalling cascade, compromised red light-induced resistance, suggesting that SA pathway played an important role in red light-induced resistance against DC3000.

## Additional files

Additional file 1: Table S1. Primers sequences used for the VIGS. Additional file 2: Figure S1. Relative spectral distribution of the LEDs. Additional file 3: Table S2. Primer sequences used for qRT-PCR validation of RNA-seq results. Additional file 4: Figure S2. Effects of DC3000 pathogen and red light alone or in combination on the light-saturated rate of CO_2_ assimilation (*A*_sat_), the maximum quantum yield of PSII (*Fv/Fm*), chlorophyll content, and electrolyte leakage in tomato leaves at 3 days after DC3000 inoculation (OD = 0.1). Additional file 5: Figure S3. Time-course of the defence-related genes transcription in tomato leaves as influenced by DC3000 infection and red light. Additional file 6: Table S3. Transcription identified to be regulated more than 2.0log_2_ fold by DC3000. Additional file 7: Table S4. Transcription identified to be regulated more than 2.0log_2_ fold by RL. Additional file 8: Table S5. Transcription identified to be regulated more than 2.0log_2_ fold by RL + DC3000. Additional file 9: Table S6. Transcription identified to be regulated more than 2.0log_2_ fold compared RL + DC3000 with DC3000. Additional file 10: Table S7. Whole genome gene expression and the annotation. The A stand for Mock, the B stand for DC3000, the C stand for RL, the D stand for RL + DC3000, B A_log_2_(Fold_change) indicated that the comparison between DC3000 and mock, C A_log_2_(Fold_change) indicated that the comparison between RL and mock, D A log_2_(Fold_change) indicated that the comparison between RL + DC3000 and mock, D B_log_2_(Fold_change) indicated that the comparison between RL + DC3000 and DC3000, D C_log_2_(Fold_change) indicated that the comparison between RL + DC3000 and RL. Additional file 11: Table S8. qRT-PCR confirmation of select transcriptions identified by RNA-seq. Additional file 12: Figure S4. Correlation analysis of gene expression values obtained from RNA-seq and qRT-PCR analysis. Additional file 13: Figure S5. Comparison of Gene Ontology (GO) classifications of gene expression in DC3000, RL, and RL + DC3000. All transcription-regulated genes were categorised into three major functional categories: biological process, cellular component, and molecular function. Additional file 14: Figure S6. Relative mRNA abundance of *NPR1*, *PI I* and *PI II* transcription in pTRV-*NPR1* and pTRV-*PI I/II* plants as compared to the pTRV vector plants. The levels were expressed as the relative values with that in control pTRV plants as 1.
