# Supplementary material for: RNA-seq analysis reveals the role of red light in resistance against Pseudomonas syringae pv. tomato DC3000 in tomato plants
Source: BMC Genomics. 2015 Feb 25;16(1):120. doi: 10.1186/s12864-015-1228-7 (PMC4349473; doi:10.1186/s12864-015-1228-7)
Supplement: Additional file 1: Table S1. — Primers sequences used for the VIGS. [file 12864_2015_1228_MOESM1_ESM.doc]

**Additional file 1: Table S1. Primer sequences used for the VIGS**

| **Gene** | **Encoded protein** | **Primer pair** |
| --- | --- | --- |
| *NPR1* | Nonexpressor of Pathogenesis-Related genes 1 | F: 5’- CGgaattcACTTCTTCGCTGATGCTAAGC-3’  R: 5’- CGggatccGACCACGGCATCAAAACTCACC-3’ |
| *PI I* | Proteinase Inhibitors I | F: 5’- CGgaattcGACTTCTTCGCTGATGCTAAGC-3’  R: 5’- CGggatccGACCACGGCATCAAAACTCACC-3’ |
| *PI II* | Proteinase Inhibitors II | F: 5’-CGgaattcATGGCTGTTCACAAGGAAGTTAATTTTGTC-3’  R: 5’-CGggatccTCACATTACAGGGTACATATTTGCCTTGGG-3’ |
