# Supplementary material for: RNA-seq analysis reveals the role of red light in resistance against Pseudomonas syringae pv. tomato DC3000 in tomato plants
Source: BMC Genomics. 2015 Feb 25;16(1):120. doi: 10.1186/s12864-015-1228-7 (PMC4349473; doi:10.1186/s12864-015-1228-7)
Supplement: Additional file 2: Figure S1. — Relative spectral distribution of the LEDs. [file 12864_2015_1228_MOESM2_ESM.doc]

**Additional file 2**

**Additional file 2:** Figure S1. Relative spectral distribution of the LEDs. P=purple light; B=blue light; G=green light; Y=yellow light; R=red light.
