# Supplementary material for: RNA-seq analysis reveals the role of red light in resistance against Pseudomonas syringae pv. tomato DC3000 in tomato plants
Source: BMC Genomics. 2015 Feb 25;16(1):120. doi: 10.1186/s12864-015-1228-7 (PMC4349473; doi:10.1186/s12864-015-1228-7)
Supplement: Additional file 4: Figure S2. — Effects of DC3000 pathogen and red light alone or in combination on the light-saturated rate of CO2 assimilation (A sat), the maximum quantum yield of PSII (Fv/Fm), chlorophyll content, and electrolyte leakage in tomato leaves at 3 days after DC3000 inoculation (OD = 0.1). [file 12864_2015_1228_MOESM4_ESM.doc]

**Additional file 4**

**Additional file 4: Figure S2. Effects of DC3000 pathogen and red light alone or in combination on the light-saturated rate of CO_2_ assimilation (*A*_sat_), the maximum quantum yield of PSII (*Fv/Fm*), chlorophyll content, and electrolyte leakage in tomato leaves 3 days after DC3000 inoculation (OD = 0.1).** The plants were kept in the dark (open column) or under red light (grey column) conditions at night without (Mock) or with the immediate inoculation of DC3000. Data are the mean ± SD of five biological replicates. Means denoted by the same letter did not differ signiﬁcantly at *p* < 0.05 according to Duncan’s multiple range test. The experiments were repeated twice with similar results.
