# Supplementary material for: RNA-seq analysis reveals the role of red light in resistance against Pseudomonas syringae pv. tomato DC3000 in tomato plants
Source: BMC Genomics. 2015 Feb 25;16(1):120. doi: 10.1186/s12864-015-1228-7 (PMC4349473; doi:10.1186/s12864-015-1228-7)
Supplement: Additional file 5: Figure S3. — Time-course of the defence-related genes transcription in tomato leaves as influenced by DC3000 infection and red light. [file 12864_2015_1228_MOESM5_ESM.doc]

**Additional file 5**

**Additional file 5: Figure S3.** **Time-course of the defence-related genes transcription in tomato leaves as influenced by DC3000 infection and red light.** The plants were kept in the dark (circle symbols) or under red light (squares symbols) conditions at night without (open symbols) or with the immediate inoculation of DC3000 (closed symbols). Data are the mean ± SD of five biological replicates with two technical replicates. Means denoted by the same letter did not differ signiﬁcantly at *p* < 0.05 according to Duncan’s multiple range test. The experiments were repeated twice with similar results.
