# Supplementary material for: RNA-seq analysis reveals the role of red light in resistance against Pseudomonas syringae pv. tomato DC3000 in tomato plants
Source: BMC Genomics. 2015 Feb 25;16(1):120. doi: 10.1186/s12864-015-1228-7 (PMC4349473; doi:10.1186/s12864-015-1228-7)
Supplement: Additional file 11: Table S8. — qRT-PCR confirmation of select transcriptions identified by RNA-seq. [file 12864_2015_1228_MOESM11_ESM.doc]

**Additional file 11: Table S8.** qRT-PCR confirmation of select transcriptions identified by RNA-seq. Relative expression levels of selected genes in the treatment of DC3000, RL and RL+DC3000. Values are shown as log_2_ ratio. The data of qRT-PCR are means of three independent biological, two technical replicates. ‘NA’ represents the missing values.

|  |  |  | **log_2_fold-change** | |
| --- | --- | --- | --- | --- |
| **Gene ID** | **Description** | **Treatment** | **RNA-seq** | **qRT-PCR** |
| Solyc12g056650 | GIGANTEA 2 | DC3000 | 2.49 | 1.66 |
|  |  | RL | 7.91 | 8.04 |
|  |  | RL+DC3000 | 7.44 | 6.86 |
| Solyc10g005080 | CCA1 | DC3000 | -0.9 | -0.72 |
|  |  | RL | -4.66 | -1.92 |
|  |  | RL+DC3000 | -1.87 | -2 |
| Solyc06g051680 | EF4 | DC3000 | 0.9 | 2.74 |
|  |  | RL | 4.33 | 2.77 |
|  |  | RL+DC3000 | 3.7 | 3.39 |
| Solyc12g057070 | UDP-glucuronosyltransferase | DC3000 | 3.21 | 2.68 |
|  |  | RL | 3.12 | 3.17 |
|  |  | RL+DC3000 | 4.5 | 4.43 |
| Solyc09g098080 | UDP-glucosyltransferase | DC3000 | 4.67 | 3.82 |
|  |  | RL | NA | 1.72 |
|  |  | RL+DC3000 | 6.06 | 3.95 |
| Solyc09g015770 | WRKY70 | DC3000 | 3.96 | 7.06 |
|  |  | RL | 0.87 | 3.97 |
|  |  | RL+DC3000 | 5.08 | 7.49 |
| Solyc08g067360 | WRKY 18 | DC3000 | 6.58 | 7.66 |
|  |  | RL | 0.13 | 2.56 |
|  |  | RL+DC3000 | 9 | 10.01 |
| Solyc10g086500 | DET2 | DC3000 | 4.15 | 6.94 |
|  |  | RL | 3.53 | 7.3 |
|  |  | RL+DC3000 | 3.66 | 7.17 |
| Solyc06g074090 | Sterol reductase | DC3000 | -3.04 | -2.8 |
|  |  | RL | 2.27 | 4.89 |
|  |  | RL+DC3000 | -3.57 | -2.28 |
| Solyc06g008590 | Auxin responsive protein | DC3000 | -4.57 | -2.39 |
|  |  | RL | -2.04 | 0.93 |
|  |  | RL+DC3000 | -5.93 | -4.21 |
| Solyc02g090890 | ZEP | DC3000 | -2.07 | -1.53 |
|  |  | RL | 3.16 | 5.06 |
|  |  | RL+DC3000 | 0.64 | 0.56 |
| Solyc01g099620 | RBOH | DC3000 | NA | 6.78 |
|  |  | RL | NA | 1.04 |
|  |  | RL+DC3000 | 6.05 | 10.52 |
