# Supplementary material for: RNA-seq analysis reveals the role of red light in resistance against Pseudomonas syringae pv. tomato DC3000 in tomato plants
Source: BMC Genomics. 2015 Feb 25;16(1):120. doi: 10.1186/s12864-015-1228-7 (PMC4349473; doi:10.1186/s12864-015-1228-7)
Supplement: Additional file 12: Figure S4. — Correlation analysis of gene expression values obtained from RNA-seq and qRT-PCR analysis. [file 12864_2015_1228_MOESM12_ESM.doc]

**Additional file 12**

**Additional file 12: Figure S4. Correlation analysis of gene expression values obtained from RNA-seq and qRT-PCR analysis.** The expression ratio (log_2_ ratio treatment/control) is presented as mean of three independent biological, two technical replicates. The Pearson correlation coefficient (r) is indicated in the figure.
