# Supplementary material for: RNA-seq analysis reveals the role of red light in resistance against Pseudomonas syringae pv. tomato DC3000 in tomato plants
Source: BMC Genomics. 2015 Feb 25;16(1):120. doi: 10.1186/s12864-015-1228-7 (PMC4349473; doi:10.1186/s12864-015-1228-7)
Supplement: Additional file 13: Figure S5. — Comparison of Gene Ontology (GO) classifications of gene expression in DC3000, RL, and RL + DC3000. All transcription-regulated genes were categorised into three major functional categories: biological process, cellular component, and molecular function. [file 12864_2015_1228_MOESM13_ESM.doc]

**Additional file 13**


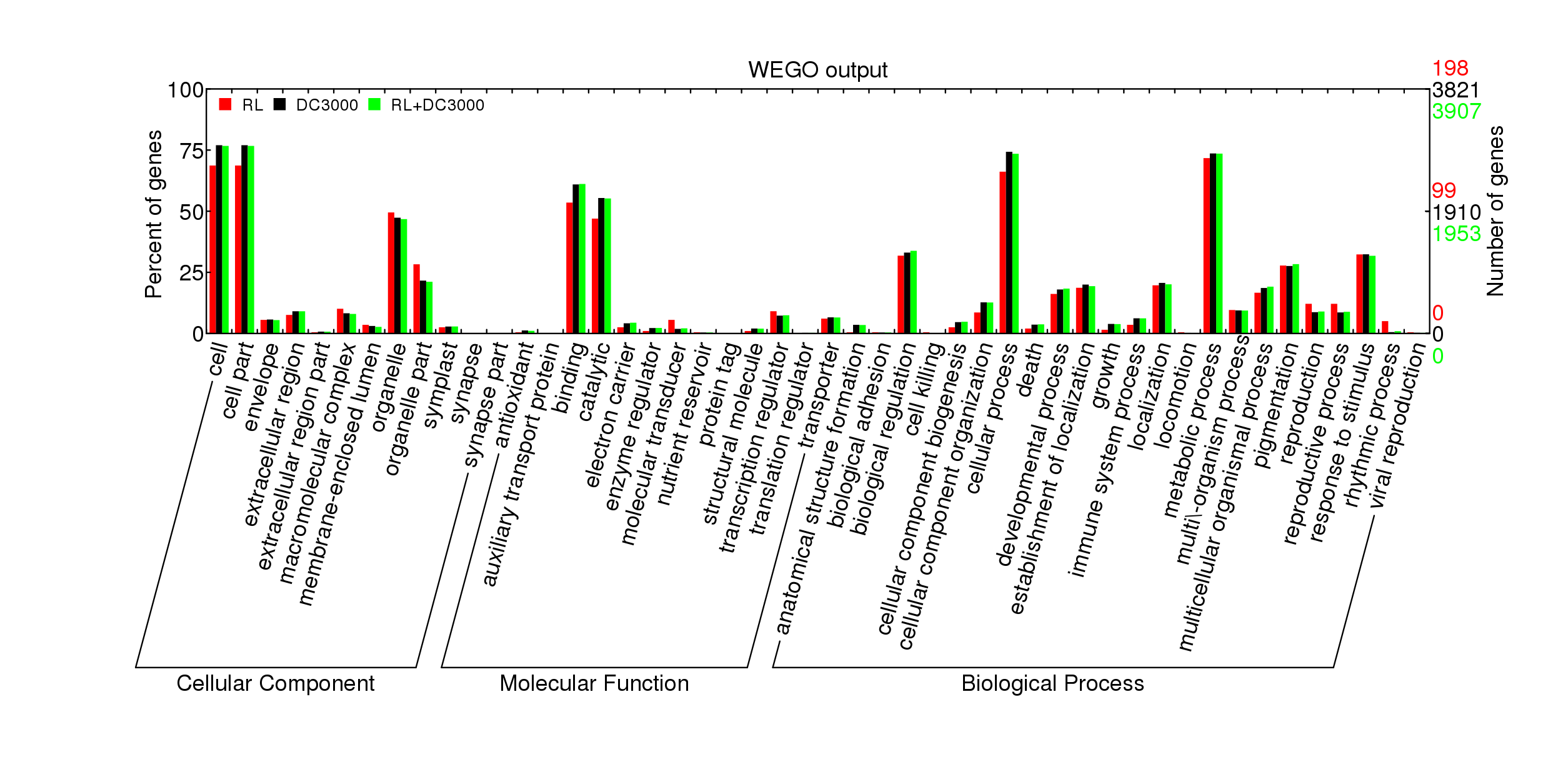


**Additional file 13: Figure S5.** Comparison of Gene Ontology (GO) classifications of gene expression in RL, DC3000, and RL+DC3000. All transcription-regulated genes were categorised into three major functional categories: biological process, cellular component, and molecular function.
