# Supplementary material for: RNA-seq analysis reveals the role of red light in resistance against Pseudomonas syringae pv. tomato DC3000 in tomato plants
Source: BMC Genomics. 2015 Feb 25;16(1):120. doi: 10.1186/s12864-015-1228-7 (PMC4349473; doi:10.1186/s12864-015-1228-7)
Supplement: Additional file 14: Figure S6. — Relative mRNA abundance of NPR1, PI I and PI II transcription in pTRV-NPR1 and pTRV-PI I/II plants as compared to the pTRV vector plants. The levels were expressed as the relative values with that in control pTRV plants as 1. [file 12864_2015_1228_MOESM14_ESM.doc]

**Additional file 14**

**Additional file 14: Figure S6. Relative mRNA abundance of *NPR1*, *PI I* and *PI II* transcription in pTRV-*NPR1* and pTRV-*PI I/II* plants as compared to the pTRV vector plants.** Data are the mean ± SD (n=20). The levels were expressed as the relative values with that in control pTRV plants as 1.
